# The suprachiasmatic nucleus at 50: looking back, then looking forward

**DOI:** 10.1177/07487304231225706

**Published:** 2024-02-16

**Authors:** Daisuke Ono, David R. Weaver, Michael H. Hastings, Ken-Ichi Honma, Sato Honma, Rae Silver

**Affiliations:** Stress Recognition and Response, Research Institute of Environmental Medicine, Nagoya University, Furo-cho, Chikusa-ku, Nagoya 464-8601, Japan; Department of Neural Regulation, Nagoya University Graduate School of Medicine, Nagoya 466-8550, Japan; Department of Neurobiology and NeuroNexus Institute, University of Massachusetts Chan Medical School, Worcester, MA 01605, USA; Division of Neurobiology, MRC Laboratory of Molecular Biology, Cambridge CB2 0QH, UK; Research and Education Center for Brain Science, Hokkaido University, Sapporo 060-8638, Japan; Center for Sleep and Circadian Rhythm Disorders, Sapporo Hanazono Hospital, Sapporo 064-0915, Japan; Department of Neuroscience and Behavior, Barnard College; Department of Psychology, Columbia University, New York City, NY 10027, Department of Pathology and Cell Biology, Graduate Program, Columbia University Medical School, New York City, NY 10032 University; Zukerman Institute Affiliate, Columbia University

**Keywords:** circadian rhythms, neuropeptides, synchronization, network, astrocyte, development

## Abstract

It has been 50 years since the Suprachiasmatic Nucleus (SCN) was first identified as the central circadian clock and 25 years since the last overview of developments in the field was published in the Journal of Biological Rhythms. Here, we explore new mechanisms and concepts that have emerged in the subsequent 25 years. Since 1997, methodological developments, such as luminescence and fluorescence reporter techniques have revealed intricate relationships between cellular and network-level mechanisms. In particular, specific neuropeptides such as arginine vasopressin, vasoactive intestinal peptide, and gastrin releasing peptide have been identified as key players in the synchronization of cellular circadian rhythms within the SCN. The discovery of multiple oscillators governing behavioral and physiological rhythms has significantly advanced our understanding of the circadian clock. The interaction between neurons and glial cells has been found to play a crucial role in regulating these circadian rhythms within the SCN. Furthermore, the properties of the SCN network vary across ontogenetic stages. The application of cell type-specific genetic manipulations has revealed components of the functional input-output system of the SCN and their correlation with physiological functions. This review concludes with the high-risk effort of identifying open questions and challenges that lie ahead.

## I Introduction: Overview of the first 25 years of the SCN (1972-1997)

In 1997, Chuck Czeisler and Steve Reppert organized a conference, held at Harvard Medical School, to celebrate the progress that had been made in our understanding of the role of the SCN in the 25 years since the initial pivotal studies demonstrating that the SCN are necessary for behavioral and endocrine rhythms. A meeting report published in the *Journal of Biological Rhythms* summarized the presentations (Weaver, 1998) and serves as a review of progress made in the first 25 years. Here, we shall briefly recap the major discoveries in the first 25 years, and will next indicate how two sets of pivotal discoveries in 1997-1998 changed the field and set the stage for explorations of the SCN in the next 25 years.

**Figure 1:**
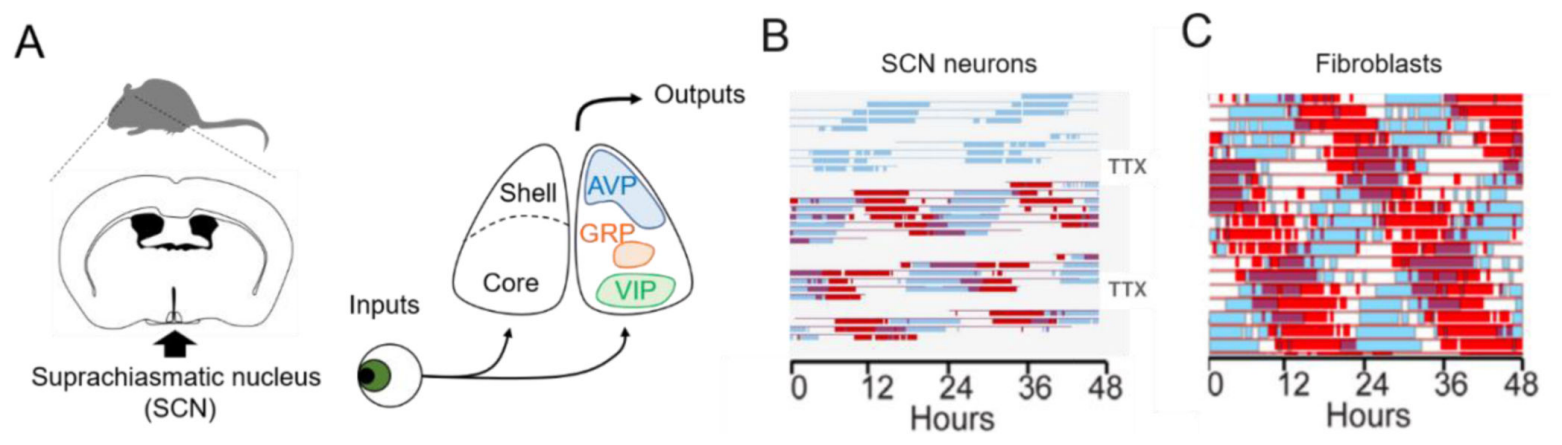
Independently phased circadian rhythms recorded *in vitro* (A) Schematic drawing of the central circadian clock, SCN. SCN neurons received light input via the retinohypothalamic tract. Several neuropeptides, such as AVP, VIP, and GRP are expressed in the SCN. Circadian information in the SCN outputs to physiology and behavior via neuronal and humoral pathways. (B, C) Profiles are double-plotted with successive days stacked on each other. Different colors represent different cells. Values over the daily average are shown as color, values below it are represented by gaps. (B) SCN neuronal electrical activity recorded from two channels of a multielectrode plate. Gaps in the recording and periods of neuronal quiescence induced by treatment with TTX are shown as gaps. Modified from Welsh et al., 1995. (C) Bioluminescence from two fibroblasts in culture. Modified from Leise et al., 2012.

In the first 25 years after its discovery as the putative biological clock in mammals (Moore and Eichler, 1972; Stephan and Zucker, 1972), most studies to define the role of the SCN started by destroying it. One noteworthy concept celebrated in the 25^th^ anniversary event was the quantitative assessment of rhythmicity and its loss after destruction of the SCN (Rusak, 1977). Numerous studies showed the SCN is *necessary* for rhythmicity in most (but not all) endpoints, species and instances (Fuller et al., 1981; Reppert et al., 1984b; Stephan et al., 1979; Weaver, 1998). The next phase of study identified rhythms within the SCN, both *in vivo* and *in vitro*, providing compelling evidence that the SCN contains an intrinsic, tissue-autonomous circadian oscillator. *In vivo* rhythms in metabolic activity were demonstrated with the then new method of ^14^C-2-deoxyglucose autoradiography (Schwartz and Gainer, 1977; Schwartz et al., 1983). These studies were extended to demonstrate circadian oscillations in fetal SCN metabolic activity (Reppert and Schwartz, 1983) and were subsequently taken *in vitro* (Newman and Hospod, 1986). Notably, in all species examined, SCN metabolic activity is higher in the daytime than at night, and light exposure at night increased SCN metabolic activity – foreshadowing studies of photic induction of immediate early genes in the late 1980s. Next came the demonstration of circadian rhythmicity in the firing rate of SCN neurons *in vivo* (Inouye and Kawamura, 1979). An intermediate step between *in vivo* and *in vitro* recordings was the demonstration in this same paper of circadian rhythms of multiunit firing from hypothalamic islands containing the SCN (Inouye and Kawamura, 1979). These authors also concluded that rhythmicity was lost in behavior and in brain regions outside the island, but this conclusion was based on a small number of animals and brain regions, and subsequent work indicated that the SCN does not need synaptic connectivity to sustain locomotor activity (Silver et al., 1996). As with metabolic activity, high levels of electrical activity occur during subjective daytime. Single-cell firing rate rhythms were subsequently identified in explants containing the SCN *in vitro* (Green and Gillette, 1982; Groos and Hendriks, 1982; Shibata et al., 1982). Rhythmic release of arginine vasopressin (AVP) from SCN explants *in vitro* (Earnest and Sladek, 1986) and its correlation with electrical activity rhythms (Gillette and Reppert, 1987) provided further evidence for the autonomy of the SCN as a circadian oscillator. Next, Welsh et al. used extracellular electrodes embedded in a culture dish to monitor electrical activity from dissociated SCN neurons for long intervals, showing that single SCN neurons have cell-autonomous circadian rhythms of firing rate (Welsh et al., 1995) (Figure 1B). The period of single-cell firing was affected by the short period *tau* mutation in dissociated hamster SCN neurons, in much the same way that the period of locomotor activity rhythms was affected by the mutation (Liu et al., 1997). Thus, a reductionist’s dream was achieved in that a property of behavioral rhythms seen in a mutant animal *in vivo* could be observed in single SCN neurons *in vitro*.

Rhythmicity of electrical and metabolic activity within the SCN and its rhythmic secretion of a neuropeptide suggest mechanisms by which the SCN could impart rhythmicity on other neural structures and thereby serve as a circadian pacemaker. Definitive evidence that the SCN functions as a circadian pacemaker was achieved through transplantation studies. Several groups succeeded in this task, showing that grafts containing the fetal hypothalamus could restore rhythmicity in rodents made arrhythmic by prior destruction of the SCN (DeCoursey and Buggy, 1989; Drucker-Colin et al., 1984; Earnest et al., 1989; Lehman et al., 1987; Sawaki et al., 1984). Importantly, Ralph et al. showed the period of restored rhythms was determined by donor tissue genotype (Ralph et al., 1990). Thus, a fundamental circadian property (period length) was dictated by the donor SCN rather than the recipient host. Finally, transplants of encapsulated grafts that prevented establishment of neural connections with the host brain demonstrated that diffusible signals were sufficient to sustain locomotor rhythms (Silver et al., 1996). Taken together, this evidence of ‘sufficiency’ in rhythm generation defined the SCN as the central circadian pacemaker in mammals.

## II On the cusp- the discovery of mammalian clock genes from 1998 onwards

Looking back, it would be nice to say that there was a wide consensus on the important questions remaining regarding the SCN at the end of the first 25 years. In fact, several discoveries were made almost immediately afterwards that seemed unpredictable just years earlier. Most of the studies cited as key for establishing the SCN as the central circadian clock in mammals above fall short of providing evidence for pacemaking function. Instead, most are consistent with showing tissue-autonomous rhythmicity – a property we now recognize to be widely distributed, with other brain areas and even numerous peripheral tissues possessing autonomous circadian oscillators. Indeed, this revolution was starting at the time of the SCN Silver Anniversary Conference. Previously, it had been largely unthinkable that oscillatory capacity was anatomically widespread. Some strategies attempting to identify a key clock molecule naively looked for rhythmic transcripts or proteins in the SCN (Chong et al., 1996; Shimizu et al., 1999). As a field, we expected rhythmicity to be restricted to the SCN, and rhythmic gene expression to be restricted to one or a few circadian clock genes that represent state variables of the clock. In fact, not all differentially expressed genes are clock genes. We now know there are thousands of rhythmic transcripts in the SCN. More importantly, we now know that the capacity for intrinsic rhythmicity is not restricted to the SCN. It is no exaggeration to say that the circadian field was turned on its head in 1998.

The first clear demonstration of oscillatory capacity of non-SCN tissue came when Balsalobre et al. reported that a serum shock could synchronize circadian rhythms of gene expression in cultured RAT-2 fibroblasts (Balsalobre et al., 1998). At the time, it seemed revolutionary to find oscillatory capacity outside the SCN and retina. Now, bioluminescent reporter genes allow detection of circadian rhythmicity from many tissues, *in vitro* and *in vivo* (Martin-Burgos et al., 2022; Nagoshi et al., 2004; Smith et al., 2022; Welsh et al., 2004; Yoo et al., 2004), and as a field we have come to take this approach, and principle, for granted. The similarity between the single-cell oscillatory behavior of SCN neurons and fibroblasts is stunning, especially when considered in the SCN-centric view of the late 20^th^ century (Figure 1B, 1C). Models in which a central brain clock imposes rhythmicity on passive peripheral tissues would soon become supplanted by the concept of the SCN as a coordinating pacemaker, entraining oscillations in numerous tissues which themselves possess the capacity for oscillations. This revised view of the capacity of other tissues to oscillate is the first major revelation referred to above.

A second major revelation was the breakthrough made in identifying the molecular mechanisms of circadian rhythm generation in mammals. By 1997, the race was on to identify genes in mammals that are homologous to those identified as important for circadian rhythmicity or period regulation in fruit flies. As databases of mammalian genomic sequences, transcripts and expressed sequence tags became available and accessible, it became possible to identify candidate ‘clock genes’ based on similarities in the deduced amino acid sequences. Database mining, low-stringency hybridization approaches and polymerase chain reaction-based methods for cloning homologous sequences within gene families and across species allowed identification of putative clock genes based on their homology to Drosophila clock components. Principles emerged: where one gene played a role in Drosophila, a family of 2 or 3 genes might play that role (or a different one) in mammals. For example, isolation of the three mammalian Period gene homologs also occurred in 1997, with several groups publishing reports of rhythmic and variably light-inducible expression in close succession (Shearman et al., 1997; Shigeyoshi et al., 1997; Sun et al., 1997). Members of basic helix-loop-helix (bHLH) transcription factor family, including Neuronal Per-Arnt-Sim (PAS) domain proteins (NPAS1-5) (Zhou et al., 1997) and Members of the PAS family (MOP1-9) (Hogenesch et al., 1997) were also identified, with important implications for clocks largely unknown at the time.

Remarkably, one circadian clock gene was identified first in mice, and second in flies. The CLOCK (Circadian Locomotor Output Cycles Kaput) mutation had been reported in 1994 (Vitaterna et al., 1994). Groundbreaking studies published in 1997 showed the mutation to be an antimorph (dominant negative), identified the genomic locus, and achieved the first transgenic rescue of a behavioral phenotype in a mammal (Antoch et al., 1997; King et al., 1997a; King et al., 1997b). Clues to the molecular function of CLOCK were initially lacking (Reppert and Weaver, 1997), but were soon supplied with the finding that CLOCK and another bHLH-PAS protein (Brain and Muscle Arnt-like protein, BMAL1, also called MOP3, Arntl, and Arnt3) (Ikeda and Nomura, 1997) could together activate transcription of per and tim and that the CLOCK mutant protein was devoid of transcriptional activity (Darlington et al., 1998; Gekakis et al., 1998). Hogenesch et al. also identified the E-box enhancer sequence through which CLOCK:BMAL1 heterodimers activate transcription (Hogenesch et al., 1998). In flies, the rhythmic expression of per transcript and protein had suggested a negative feedback loop as the cellular mechanism underlying circadian rhythmicity (Hardin et al., 1990). In a very short time, the homologous structure of the fly and mammalian circadian clocks was recognized, and indeed inclusion of Neurospora in the conversation indicated that a transcriptional-translational feedback loop was THE cellular basis for circadian rhythmicity, even when the molecules involved were not structurally homologous (Dunlap, 1999). Elucidation of the molecular mechanisms of circadian rhythms in Drosophila led (with a lag of twenty years) to Jeffrey Hall, Michael Rosbash and Michael Young receiving the 2017 Nobel Prize in Physiology or Medicine (Sehgal, 2017).

## III Methodological advances in the second 25 years:1998-2022

**Figure 2:**
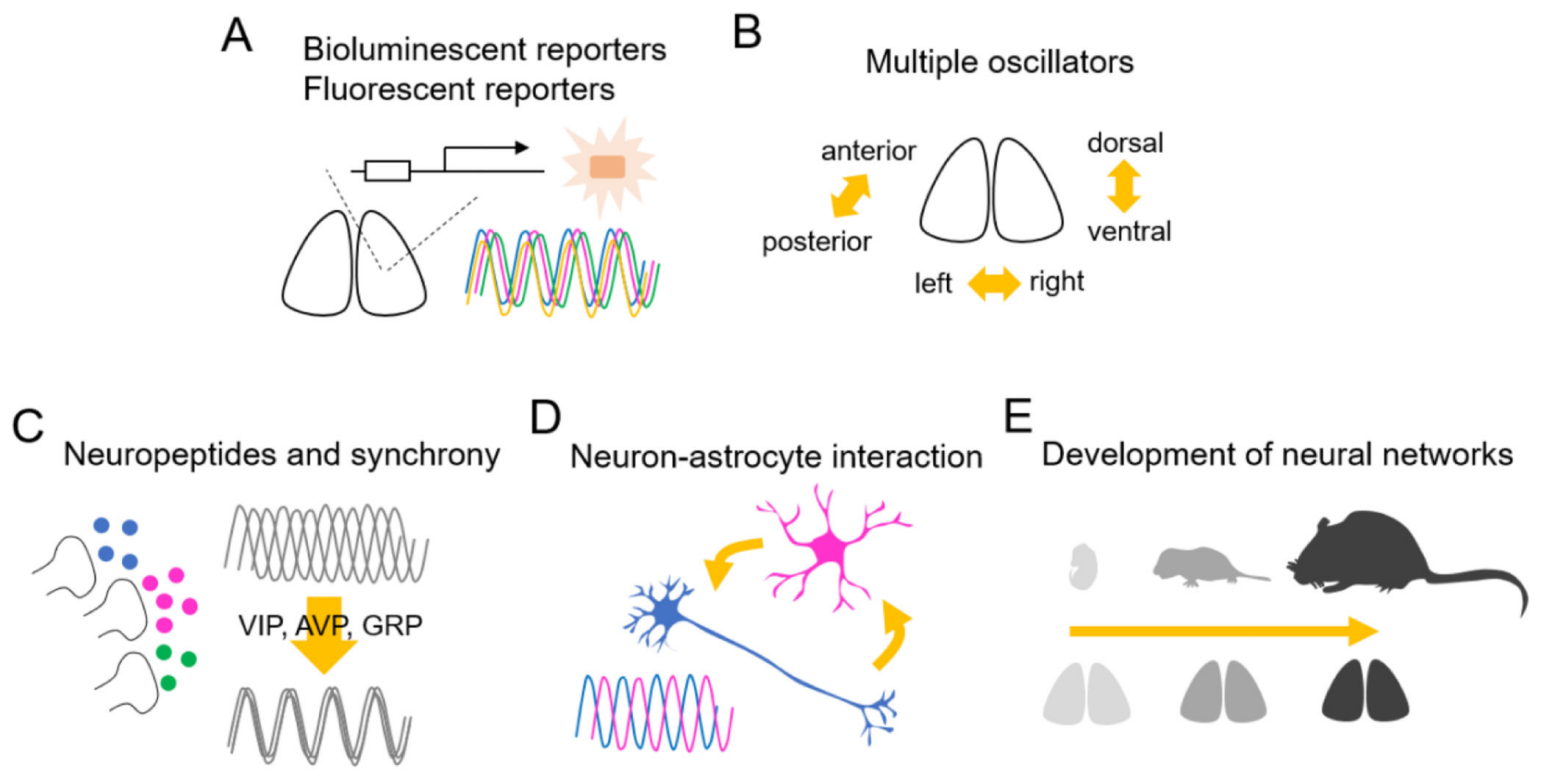
Fundamental aspects of the neuronal circuits of the SCN discovered in the second 25 years (A) Bioluminescent and fluorescent reporters provided many aspects of the cellular as well as circuit-level function of circadian rhythms in the SCN. (B) The SCN includes multiple circadian oscillators responded to by environmental light-dark conditions, which could regulate the onset and offset of circadian behavior. (C) Synchronization of cellular circadian rhythms in the SCN is regulated by neuropetidergic signaling, such as VIP, AVP, and GRP. (D) Circadian rhythms are observed in both neurons and astrocytes in the SCN, and the interaction of neurons and astrocytes regulates circadian rhythms in the SCN. (E) Neural networks in the SCN are developed depending on age.

Research in the first 25 years used tools such as lesions and *in vivo* and *in vitro* measures of metabolic and electrical activity. With the advent of clock genes, methodological advances after the mid-1990’s drove new research in the study of the SCN (Figure 2A). One of the areas to progress was the field of genomics, with data on genes and transcripts becoming readily available. As noted above, this enabled the hunt for mammalian homologues of clock genes identified in *Drosophila* and allowed higher-throughput determination of gene expression. Initially this involved microarrays and proceeded to the present day when methods available include whole-genome and whole-transcriptome sequencing, single-cell sequencing, and sophisticated methods for identifying where transcription factors and specific modifications of histone proteins occur throughout the genome (chromatin immunoprecipitation). In parallel, computational approaches have improved, allowing assessment of rhythmicity at the whole-transcriptome level. These advances have benefitted every field of biology, and have contributed greatly to our understanding of the molecular basis of the circadian clock.

Advances in approaches to manipulate the mouse genome have made important contributions to the circadian field. The role of each of the putative circadian clock genes was examined by generating whole-body knockout mice using the now-antiquated approach of generating embryonic stem (ES) cells in which homologous recombination had deleted a key region of the targeted locus. More contemporary gene editing approaches such as TALENs and CRISPR/Cas9 are much more efficient and are not limited to mice as was the ES cell approach. In addition, the field has adopted tools for more cell-type specific gene manipulation, with numerous clock genes now available as conditional alleles, in which the lox recognition sequence for Cre recombinase has been introduced to specific loci (called flanked by lox or floxed), allowing excision of the flanked DNA sequence. Numerous lines of mice carrying Cre recombinase under control of a cell-type specific promoter sequence have been generated to facilitate intersectional deletion of alleles of interest.

In addition, pairs of different Cre-sensitive floxing sequences in the proper orientation can flip the orientation of the floxed DNA. This double-floxed Inverted open reading frame (DIO, also called FLEX) approach is extremely useful in combination with adeno-associated viral (AAV) vectors injected to specific neural sites. Inversion of the sequence leads to expression of the cargo only in the local neuronal population expressing Cre recombinase. Reporter alleles have been generated in which Cre recombinase activity leads to excision of one reporter and expression of another (Shan et al., 2020; Smith et al., 2022). Specific populations of neurons can be made to express the target molecules for optogenetic or chemogenetic activation or inhibition in a Cre-dependent manner, allowing reversible manipulation of neuronal activity within specific neuronal populations (Brancaccio et al., 2013; Fan et al., 2015; Jones et al., 2015). Molecules useful for tract tracing, including trans-synaptic tract tracing, can be specifically expressed using the FLEX approach. Finally, specific neuronal populations can be labeled (Guenthner et al., 2013) or killed (Saito et al., 2001) using Cre-based or tetracycline-regulated approaches. Doxycycline-regulated gene expression with the Tet Operon can produce cell-type specific expression of genes of interest that is readily reversible (Hong et al., 2007). Similarly, translational switching allows reversible expression at the protein level (Maywood et al., 2018).

With the identification of rhythmically expressed genes (including clock genes) came the desire to use these genetic sequences to create reporters of rhythmicity (see Smith et al., 2022). Promoter sequences of rhythmically expressed genes were used to generate bioluminescent and fluorescent reporter constructs which have been used to monitor circadian rhythms in single SCN cells *in vitro* and *in vivo*, and in non-SCN cells and tissues (Abe et al., 2002; Kuhlman et al., 2000; Nagoshi et al., 2004; Welsh et al., 2004; Yamaguchi et al., 2003; Yamazaki et al., 2000; Yoo et al., 2005; Yoo et al., 2004). These reporters have contributed greatly to our understanding of the interactions among neurons in the SCN and in defining the molecular mechanisms of circadian oscillations, as discussed below (Figure 2A). These reporters have been used in the context of transgenic and knock-in rodent models as well as following their introduction by viral vectors (e.g., AAV, adenovirus, or lentivirus). The use of luciferase reporters with a range of excitation/emission spectra has allowed multimodal imaging, and has also been combined with assessment of neuronal activity such as combined bioluminescence and fluorescence (Brancaccio et al., 2013) or calcium fluorescence and electrical activity (Ono et al., 2017).

These *in vitro* approaches have been complemented by new methods of *in vivo* recording of circadian rhythms, including using an optical fiber to record SCN clock gene and Ca^2+^ dynamics in behaving animals (Jones et al., 2018; Mei et al., 2018; Yamaguchi et al., 2001). In addition, intersectional methods have enabled (Saini et al., 2013; Shan et al., 2020; Sinturel et al., 2021; Smith et al., 2022) long-term monitoring of bioluminescence rhythms of specific tissues (e.g., liver) with minimally invasive approaches (Katsioudi et al., 2022; Martin-Burgos et al., 2022; Tahara et al., 2012). These have already begun to illuminate the relationship between SCN and peripheral oscillators, a critically important factor in metabolic health and disease.

In terms of circadian neuroanatomy, tissue-clearing methods that allow 3D imaging of the intact SCN (Renier et al., 2014; Susaki et al., 2014) have provided a comprehensive view of morphological relationships among cell groups (Wen et al., 2020) and have also revealed that the SCN has a portal system similar to the pituitary portal system (Yao et al., 2021). Understanding the connectivity of identified cell groups has been enhanced by intersectional uses of conditional anterograde and mono-synaptic retrograde rabies tracing (Todd et al., 2020; Yuan et al., 2018). It is likely that this will lead to a far richer wiring diagram of SCN-brain connectivity with relevance to the question of how the SCN controls a diversity of physiological and behavioral outputs with different phases: where does temporal granularity arise? One answer to this will come from deeper understanding of SCN cell-types, acquired, for example, by single cell RNA sequencing (scRNAseq) (Morris et al., 2021; Todd et al., 2020; Wen et al., 2020). With transcriptionally based approaches we can now explore what these cell groups do within the SCN and what they do to downstream targets.

Having described systems, it is then imperative to manipulate them. High-throughput chemical screening (Hirota and Kay, 2009; Hirota et al., 2010; Tamai et al., 2018) has the potential to identify clock component and modulators, but is not readily amenable to assessing SCN function. Furthermore, although RNA interference has been widely used in assessing circadian rhythms in Drosophila and mammalian cell culture models (Axelrod et al., 2015; Maier et al., 2021; Maier et al., 2009; You et al., 2018; Zhang et al., 2009) it has been used rarely in the SCN (Gavrila et al., 2008; Hermanstyne et al., 2017; Ikeda and Ikeda, 2014). The exciting developments in CRISPR-based gene editing may offer the flexibility, capacity and specificity to interrogate SCN functions in completely new dimensions.

Ever since Pittendrigh’s breakthrough study, modeling work has had a key role in unraveling the nature and function of oscillators (Pittendrigh, 1960). In the past 25 years the availability of tools that allow tracking of gene and protein expression and electrical activity over time, sometimes simultaneously, have provided opportunities to analyze the SCN’s complex, multiscale spatiotemporal environment. The ready availability of powerful computers has led to increasing and more sophisticated use of mathematical modeling to interpret and to guide empirical research on the SCN’s network structure (Abel et al., 2016; Antle et al., 2003; Antle et al., 2007; DeWoskin et al., 2015; Jeong et al., 2016; Kim et al., 2005; Pauls et al., 2014; Vasalou et al., 2009; Yoshikawa et al., 2021). Providing a rich resource for modeling work in the past 25 years, many studies have made high-throughput time-series data available on online resources such as CircaDB, SCNseq and RhythmicDB (Castellana et al., 2022; Pembroke et al., 2015; Pizarro et al., 2013).

## IV From Silver to Gold: SCN in the second 25 years, 1998 to 2022

### IV A Overview of the starting point

In the classical model, the SCN is viewed as bearing an input, a clock, and an output, and this presents a framework for organizing our consideration of developments in this field in the 25 years following the discovery of the SCN. Within the SCN, the identification of distinct core and shell SCN subregions set the stage for more detailed analysis of SCN inputs, as these subregions are anatomically different and have distinct functions (Hamada et al., 2001; Miller et al., 1996; Miyake et al., 2000). On the SCN input side, the major focus was on photic cues that travelled to the SCN via the retinohypothalamic tract (RHT) and non-photic cues that reached the SCN via the inter-geniculate leaflet (IGL). Non-photic signaling via the SCN was also carefully delineated (Hastings et al., 1997). Anatomical studies involving injection of retrograde tracer into the SCN (and in some cases, anterograde confirmation), suggested that the SCN afferent input was extensive, but Moga and Moore noted that only three inputs, namely from the retina, the midbrain raphe, and the IGL were well characterized (Moga and Moore, 1997; Vrang et al., 1995). On the SCN output side, early anatomical studies had identified key aspects of SCN organization and efferent connections in hamsters and rats. However, the projection from the SCN to the pineal gland was the only fully characterized efferent pathway, and pineal melatonin had been recognized early on as an important hormonal signal regulated by the SCN (Weaver, 1998). The summary by Moore (1996) captured the status of knowledge of the time: “The output of the suprachiasmatic nuclei is quite restricted but becomes amplified by a set of downstream components of the system that appear to provide a widespread circadian signal.” (Moore, 1995). Despite knowledge of these downstream relay pathways, it was mysterious after 25 years of SCN research (Weaver, 1998) as to how this small population of small neurons could possibly signal time to the body.

### IV B Revealing SCN organization

The SCN has two distinct subdivisions: the dorsal region is characterized by AVP-containing neurons and the ventral region characterized by vasoactive intestinal polypeptide (VIP) and gastrin releasing peptide (GRP) containing neurons (Moore et al., 2002). The ventral region receives retinal innervation, while the dorsal region is primarily associated with more robust rhythmicity and extra-SCN outputs (Hamada et al., 2001) (Figure 1A). Diverse neuropeptides are expressed in the SCN, and recent studies have attempted to define the role of these subpopulations and circuits (see below).

A number of studies suggest circadian oscillations with different intrinsic periods in the dorsal and ventral SCN regions, for example being shorter in the dorsal region than the ventral (Noguchi et al., 2004), resulting in an advanced phase of the dorsal relative to the ventral SCN (Yan and Okamura, 2002). The two oscillators desynchronized spontaneously (Shinohara et al., 1995) upon TTX treatment (Enoki et al., 2012), surgical separation (Koinuma et al., 2013), abrupt phase-shift of LD cycle (Nagano et al., 2003) or by exposure to constant light (LL) (Yan et al., 2005). Functionally distinct oscillations were also observed in the anterior and posterior regions of the SCN (Bedont et al., 2018; Inagaki et al., 2007; Jagota et al., 2000; Noguchi and Watanabe, 2008), which may reflect the 3-dimensional anatomy of shell wrapping around core at the rostral and caudal poles of the SCN. Studies of dispersed SCN neurons also indicate heterogeneity of circadian period among the cellular oscillators. The period of single-cell rhythms ranged from 20 h to 28 h, when studying either neuronal activity (Herzog et al., 1998; Honma et al., 1998; Welsh et al., 1995) or bioluminescence (Liu et al., 2007). The distribution of periods was more restricted in SCN slice culture and was very narrow in circadian locomotor activity rhythms (Herzog et al., 1998; Honma et al., 2004). These results indicate that the circadian periods of single-cell oscillations are diverse and reveal the importance of cell-to-cell communication within the neural network leading to coherent and precise circadian rhythms in the SCN. Considerable progress has been made in understanding these mechanisms (see “Circuits” section below).

### IV C SCN Inputs and Outputs

In the 1998-2023 era, interest in the input and output pathways of the SCN expanded substantially. The understanding of molecular mechanisms of the SCN clock, along with the development of new tools reinvigorated exploration of SCN inputs and outputs (Krout et al., 2002; Starnes and Jones, 2023). Much of this research focused on mice (due to their genetic tractability), and increasingly, attention turned to the contributions of volume transmission.

#### IV C 1 SCN neural inputs

On the input side, the discovery of intrinsically sensitive retinal ganglion cells (ipRGCs) (Provencio *et al*. 2000) not only solved a longstanding question about the role of classical photoreceptors in circadian entrainment but also significantly refined our understanding of how photic information reaches the brain (Schmidt et al., 2011). Mapping of afferent input to the SCN of diurnal animals set the stage for better understanding of the functions of various clock inputs and the impact of exercise (Ni et al., 2021; Yan et al., 2020). Wheel-running activity feeds back to the circadian system and changes its intrinsic period. Wheel-running even reverses the phase of activity in Degus and Nile grass rats, changing the activity profile of a subset of animals from diurnal to nocturnal (Bano-Otalora et al., 2021; Blanchong et al., 1999; Yan et al., 2020). More broadly, non-photic inputs can either enhance or block photic inputs (Yannielli and Harrington, 2004; Yannielli et al., 2004) leading to studies of application. For example, exercise can restore circadian function lost with age (Leise et al., 2013). Importantly, this work goes beyond the idea that light acts as the dominant or only Zeitgeber, by showing that effects of light can be modified by lifestyle (exercise).

A more nuanced picture has emerged of a heterogeneous population of neurons in the SCN clock, sensitive to light but also to signals such as hormonal cues (Karatsoreos, 2019; Karatsoreos and Silver, 2007; Mong et al., 2011) and thermal signals (Abraham et al., 2010; Bordyugov et al., 2015; Herzog and Huckfeldt, 2003; Ruby et al., 1999). In mammals, light provides the primary input to the SCN, and here it is integrated with non-photic zeitgebers (Ashton et al., 2022). The mechanism by which light entrains the clock and produces advances and delays can be detected in regional responses within the nucleus (Kim and McMahon, 2021). It was already known that the SCN receives mono- and multi-synaptic input from numerous brain regions. Furthermore, non-photic inputs to the SCN were also reported. For example, NPY release from IGL neurons and serotonin release from dorsal or median raphe nucleus produced phase advance shifts of SCN circadian rhythms (Mrosovsky, 1996). Dopamine neurons in the ventral tegmental area have inputs to the SCN and accelerate photoentrainment (Grippo et al., 2017). Cholinergic neurons from the basal forebrain to the SCN have been suggested as input pathway (Todd, 2020). Since 1997, significant effort was directed at understanding the import of the SCN’s cellular diversity. Increasingly, information on afferent input to specific SCN neuronal subtypes has become available, though most research has been directed at the best known SCN peptides, VIP and AVP with some attention to neurons expressing GRP, cholecystokinin (CCK) and somatostatin (SST).

It has long been known, based on conventional tracing techniques, that dense retinal inputs from the RHT reach the VIP-containing region of the core SCN (Card and Moore, 1991). The reinvigorated anatomy opened the door to functional studies of specific SCN populations. The availability of new tools to study specific populations fine-tuned our understanding of SCN heterogeneity. For example, classification of VIP cell types based on the nuclear mRNA profiles of single cells pointed to two different functional subtypes: those that express VIP and GRP or those that express VIP alone. The existence of two different populations of VIP neurons has been further confirmed by single-cell RNA sequencing method (Todd et al., 2020; Wen et al., 2020) and by evidence that there are differences in firing patterns among VIP neurons in the SCN (Collins et al., 2020; Mazuski et al., 2020). These findings map well to earlier work based on conventional immunochemical analysis that pointed to the same two topographically and functionally distinct sub-groups of VIP neurons (Kawamoto et al., 2003; Romijn et al., 1998). In the case of CCK, a rabies virus- and Cre/loxP-based, cell type-specific, retrograde tracing system has provided a map of whole-brain monosynaptic inputs to SCN CCK neurons (Yuan et al., 2018).

These anatomical studies set the stage for exploring the functional significance of these numerous afferent inputs by examining the behavior of distinct populations of neurons *in vivo* (Davidson et al., 2023). For example, work using *in vivo* fiber photometry over multiple days demonstrates that VIP neurons are spontaneously rhythmic and are necessary for responding to photic cues that reset circadian timing (Jones et al., 2018). Comparison of thresholds at three wavelengths of wheel-running rhythms, masking and the pupillary light reflex indicate that dim light can entrain circadian rhythms even when it fails to produce more easily measurable acute responses to light such as phase shifting and melatonin suppression (Butler and Silver, 2011). AVP neurons lie in the SCN shell and receive significant afferent input from core neurons (Romijn et al., 1997; Varadarajan et al., 2018). In *vivo* investigations of rhythmic behavior of AVP neurons using miniaturized calcium microscopy and optogenetically targeted single-unit activity recordings indicate that while AVP neurons are important for organismal rhythmicity, individual cellular rhythms are unstable and diverse, exhibiting temporal and spatial heterogeneity (Davidson et al., 2023). For CCK, tract tracing and immunochemistry analyses indicate that these neurons do not respond to photic cues but get direct input from numerous brain regions (Hannibal et al., 2010; Yuan et al., 2018) though little is known of the functional significance of this peptidergic population in SCN network organization. Finally, while the peptidergic phenotype of the neurons was not determined there is evidence that some individual neurons in the mouse SCN are part of both input and output pathways, providing a direct link for photic inputs to influence neural targets (De la Iglesia and Schwartz, 2002).

#### IV C 2 Diffusible inputs to the SCN

The possibility that systemically circulating gonadal hormones might affect rhythmicity was first suggested in the work of Daan et al. (Daan et al., 1975). Clues as to the underlying mechanisms emerged with the demonstration that the SCN bears androgen and estrogen receptor-containing neurons in spatially segregated populations (Abizaid et al., 2004; Karatsoreos et al., 2011) and that there are dose-dependent effects of androgens in circadian responses to light (Butler et al., 2012a). Finally, as noted in his review (Belle, 2015), “… in all animal species studied thus far, including humans, high-affinity receptors for melatonin, estrogen, androgen and progesterone are present in the SCN. These hormones can act to modulate the electrical activity of SCN neurons and adjust the phase of the SCN clockworks”. Humoral inputs to the SCN are not limited to secretions of glandular origin. The choroid plexus, a circumventricular organ (CVO), is the source of a diffusible signal that modulates the free-running period of the SCN clock, likely via circulation in the cerebrospinal fluid (CSF) (Myung et al., 2018). Finally, the role of melatonin on the SCN and on circadian timing has been much examined (Dubocovich et al., 1996; Pevet, 2016), although its physiological role as an input to the SCN is obscure in rodents (Weaver, 1999).

#### IV C 3 SCN neural outputs

While SCN outputs remain relatively less studied than its inputs, there have been significant advances in understanding SCN targets and signaling via both synaptic and volume transmission. Initial observations that the SCN projects sparsely and locally to a few nearby adjacent hypothalamic regions have held up over time. At the anatomical level, attention has turned to questions of the SCN topographical organization and the distinct neurochemical composition of these connections. At the behavioral and physiological level, the evidence for a contribution by the SCN in controlling neuroendocrine rhythms, water intake, sleep quality, glymphatic circulation, body temperature, susceptibility to inflammation, efficacy of timing of cancer drugs and more, has grown exponentially (Kalsbeek et al., 2006; Starnes and Jones, 2023). A hierarchy of SCN neurochemical output signals with substantial functional redundancy can contribute to these multiple effects (Maywood et al., 2011).

Anatomical studies using anterograde and retrograde tracing protocols suggest that all SCN targets receive convergent information from both the light-induced and rhythmic neurons of the SCN, albeit to varying degrees (Kriegsfeld et al., 2004). A lovely demonstration of SCN signaling to target regions was the demonstration of antiphase expression in the right and left side of the hamster SCN and its relation to the secretion of luteinizing hormone (LH) (de la Iglesia et al., 2003). It was subsequently demonstrated that in these split hamsters, the arrangement of oscillation in the bilateral SCN involves a 4-way split in 24-h rhythms of FOS. It is not only the right and left side of the SCN that are in antiphase, but also the core and shell regions within each SCN are in antiphase (Tavakoli-Nezhad and Schwartz, 2005; Yan et al., 2005). In a follow-up study aimed at understanding when SCN neurons send time-setting signals to monosynaptic targets in neurosecretory neurons, Butler et al. measured wheel-running and FOS expression in the brains of split and unsplit hamsters housed in constant light and in controls housed in a light–dark cycle. In all conditions studied, the onset of FOS expression in monosynaptic neurosecretory target sites occurred at a common phase reference point of the daily oscillation in the SCN, suggesting that each SCN may signal to these targets once daily (Butler et al., 2012b).

Taken together, the foregoing studies point to regional specializations, and lead to the hypothesis that separate SCN subpopulations provide distinctly different signals to control specific rhythms in physiology and behavior. Consistent with this possibility, AVP efferents to the OVLT are implicated in anticipatory drinking (Gizowski et al., 2016). Conversely, GABA neurons in OVLT are an input to SCN regulating drinking behavior, suggestive of a feedback loop (Gizowski and Bourque, 2020). Another major output of the SCN is to the paraventricular nucleus of the hypothalamus (PVN). A multi-synaptic pathway implicates VIP efferents in the control of heart rate and corticosterone secretion (Jones et al., 2021; Paul et al., 2020), while PVN-directed SCN outflow regulates autonomic functions, including melatonin production (Kalsbeek and Buijs, 2021; Kalsbeek et al., 2006; Moore and Danchenko, 2002). AVP of SCN origin has been implicated in autonomic system regulation, notably the corticosterone rhythm (Kalsbeek et al., 2010) and an SCN-PVN-Lateral hypothalamus pathway is involved in circadian-regulated wakefulness in mice (Ono et al., 2020).

The most well-defined SCN output pathway leads to the regulation of arylalkylamine amino transferase (AANAT) activity in the pineal gland. AANAT is the rate-limiting enzyme in melatonin synthesis, and is induced at night with a high-amplitude rhythm by noradrenergic signaling (Klein et al., 1997). The SCN projects to the paraventricular nucleus of the hypothalamus to influence autonomic function. Fibers from the PVN impinge upon preganglionic fibers in the spinal cord, which project to the superior cervical ganglion, which ultimately innervates the pineal. The GABAergic projection from the SCN to the PVN suppresses AANAT activity. Daytime inhibition of GABA signaling in the PVN leads to increased AANAT activity, as does destruction of the SCN (Kalsbeek et al., 2000). Thus, SCN output actively inhibits AANAT activity during the daytime, and its quiescence at night allows the nocturnal increase, mediated by noradrenergic signaling (Klein et al., 1997). Interestingly, the pineal melatonin rhythm peaks at night, irrespective of whether a species is day- or night-active. Thus, downstream effectors of other autonomic and endocrine rhythms diverge in their phase, but how this is achieved remains unclear.

The bulk of SCN efferents project to the sub-paraventricular zone (SPZ) of the hypothalamus, from which they disperse to other sites (Deurveilher and Semba, 2005). Lesions of the ventral SPZ disrupt rhythms of sleep and locomotor activity, while lesions of the dorsal SPZ disrupt the body temperature rhythm (Abrahamson and Moore, 2006; Lu et al., 2001). Chou et al. subsequently found that the dorsomedial hypothalamus is a key locus in the regulation of behavioral rhythms, including sleep, food intake and locomotor activity, due to projections it receives via the SPZ (Chou et al., 2003).

#### IV C 4 Diffusible outputs from the SCN

A different development with regard to SCN output signals came about from SCN transplant studies (LeSauter and Silver, 1998; Ralph and Lehman, 1991). SCN transplants into the ventricular systems of the brain restore locomotor activity rhythms, even when the recipient is a mutant lacking endogenous oscillators (Sujino et al., 2003). Silver et al. demonstrated unequivocally that diffusible signals from the SCN were sufficient to support behavioral rhythmicity by placing the transplant within a copolymer capsule that allowed diffusion but blocked neural efferents (Silver et al., 1996). Furthermore, co-culture of adult SCN slices from *Cry*^*-/-*^*;Cry2*^*-/-*^ mice (recipients) with wild-type, neonatal donor SCN slices restored the circadian rhythm in PER2::LUC expression from the recipient slice (Maywood et al., 2011; Ono et al., 2013). GABA could play a role in circadian outputs (Maejima et al., 2021; Ono et al., 2019; Ono et al., 2020; Paul et al., 2020). Also, the neuropeptide *Prokineticin 2* (*Prok2*) may be an important mediator of the circadian control of physiology and behavior by SCN efferents (Cheng et al., 2002; Lambert et al., 2005; Prosser et al., 2007). Other proposed diffusible output signals regulating locomotor activity include CLC and TGF (Kramer et al., 2001; Kraves and Weitz, 2006). Mass spectrometry-based analysis revealed that many additional neuropeptides, which hypothetically are candidate output factors, can be released from the SCN (Hatcher et al., 2008).

While the transplant work proved the efficacy of diffusible signals in sustaining locomotor rhythmicity, it did not reveal the identity of the diffusible signal(s), nor the target site(s), nor the route by which it travelled to reach the target(s). A start to address those questions comes with the finding of a portal pathway between the SCN and the OVLT (Yao et al., 2021). This may open the next era of research into the vascular system whereby circadian signals reach the brain as the study of diffusible signaling is in its infancy. Diffusible signals that presumably course from the capillary bed of the SCN to the capillary bed of the OVLT, with its leaky blood vessels, present the same opportunities, all of which remain to be investigated. A prominent AVP rhythm exists in CSF (Schwartz and Reppert, 1985) and may be a complementary route of influence over circumventricular structures. Although there is evidence of diffusible outputs from the SCN, it remains to be determined how the SCN utilizes volume transmission, either locally or globally, to coordinate daily rhythms in physiological or behavioral rhythms.

### IV D SCN cells and circuits

#### IV D 1 Cell-autonomous vs. network-level oscillations

The first 25 years indicated that individual SCN neurons show intrinsic circadian rhythms. During the second 25 years, using manipulation and imaging methods the field has further identified not only cellular functions but also molecular mechanisms of networks in the SCN. While SCN neurons exhibit circadian rhythms in firing frequency in dispersed cell culture (Welsh et al., 1995), intercellular communications might still be involved in this culture condition. Webb et al. physically isolated single SCN neurons in a dish and measured PER2::LUC rhythms and found that circadian rhythms were still observed in some isolated single cells (Webb et al., 2009). They also reported that circadian rhythms are intrinsic to VIP, AVP, and other SCN neurons. They next asked whether there is a specialized class of intrinsically circadian neurons within the SCN. To answer this, they measured cellular PER2::LUC rhythms in the SCN slice and first applied tetrodotoxin (TTX) to block inter-cellular communications, and then washed it out. They repeated this experiment two times using the same SCN slice. Interestingly, SCN cells that showed circadian rhythms during the first TTX application did not always show circadian rhythms during the second TTX application. These results suggest that neurons throughout the SCN are capable of cell-autonomous circadian rhythm generation, but that the expression of rhythmicity is stochastic. In dispersed cell cultures of the SCN, the majority of cells showed circadian PER2::LUC rhythms, but some showed Ca^2+^ rhythms (Noguchi et al., 2017). On the other hand, almost all cell shows robust circadian rhythms of PER2::LUC, *Per1-luc, Bmal1-Eluc*, Ca^2+^, and spontaneous firing in SCN slices (Enoki et al., 2012; Liu et al., 2007; Myung et al., 2012; Nakamura et al., 2002; Yamaguchi et al., 2003). Simultaneous recordings of these parameters revealed that the circadian periods of *Per1-luc* and *Bmal1-ELuc* were slightly different suggesting the presence of at least two circadian pacemakers in the SCN, with different molecular mechanisms (Ono et al., 2017).

The mechanisms underlying neuronal circuitry within the SCN also have been examined. The period distribution and cycle-to-cycle variability of SCN cellular circadian rhythms in dispersed cells was greater than for cells in intact SCN slices (Herzog et al., 2004; Honma et al., 2004). This indicated that the synchronization and temporal precision of cellular circadian rhythms in the SCN are regulated by neuronal networks. The importance of cellular networks for the robustness of circadian rhythms in individual SCN cells against genetic perturbation was shown by measuring PER2::LUC rhythms from SCN cells in dispersed cells and organotypic slices from CRY1-deficient mice (Liu et al., 2007). Whereas only a few individual SCN cells showed circadian oscillation in dispersed cell culture, very stable and synchronized cellular circadian oscillations were observed in the SCN slice. This suggested that intercellular networks in the SCN are able to stabilize and synchronize the cell-autonomous circadian oscillators, and thereby compensate for genetic deficiency (Liu et al., 2007).

#### IV D 2 Mediators of network-level oscillations

The SCN contains a number of neuropeptides and neurochemicals involved in the cell-to-cell communications and neurotransmission (Moore, 2013). The networks of SCN neurons containing these diverse phenotypes have been extensively studied (Varadarajan et al., 2018). Among them, VIP and AVP are the two best studied peptides involved in network transmission. VIP signaling is required for the synchronization of cellular circadian rhythms (Figure 2C). VIP or VIP receptor 2 (*Vipr2*) KO mice exhibited deteriorated behavioral rhythms under constant darkness, and cellular rhythms were desynchronized in the SCN (Aton et al., 2005; Colwell et al., 2003; Harmar et al., 2002; Maywood et al., 2006). Daily application of VIP in culture medium synchronized cellular circadian rhythms in VIP-null SCN. AVP and GRP are also critical for the synchronization of cellular circadian rhythms in the SCN, an effect also seen with potassium-induced depolarization. Maywood et al. developed a co-culture technique to explore further the role of neuropeptides in SCN circadian timekeeping (Maywood et al., 2011). They measured PER2::LUC rhythms from the SCN in *Vip* KO mice and then placed on top a WT SCN slice, which lacked the reporter. The amplitude of PER2::LUC rhythms and the cellular synchrony of the *Vip* KO SCN increased after co-culturing of WT SCN, even when a molecular weight cut-off membrane separated the tissues. This was indicative of paracrine activation by VIP released by the WT SCN on to the mutant slice. A similar effect was seen when *Vipr2*-null SCN were co-cultured with a WT SCN, indicative of other, VIP-independent synchronizing cues. When AVP or GRP receptor antagonists were applied to the co-culture medium, graft-dependent circadian amplitude was attenuated. Inhibition of AVP receptors, V1a and V1b, altered circadian periods and phases of regional oscillating cells differentially and the disruption of both receptors *in vivo* allows instantaneous resetting of activity in response to shifting of the light-dark cycle (Yamaguchi et al., 2013). These results indicate that AVP or GRP are also important in sustaining cellular networks in the SCN (Figure 2C).

VIP neurons are considered essential for the normal light-mediated resetting of the SCN circadian system (Jones et al., 2018). Mice lacking VIP did not adapt to short and long photoperiods (Lucassen et al., 2012). This suggests that VIP signaling is essential for the adaptation of circadian rhythms to changes in day length. When VIP SCN neurons were optogenetically stimulated to mimic a long photoperiod, it had similar effects on behavior to a true extension of the photoperiod (Tackenberg et al., 2021). The number of VIP-expressing neurons in the SCN increased under long photoperiods and decreased under short photoperiods (Porcu et al., 2022). This suggests that VIP neurons are sensitive to day length changes and may play a role in encoding the seasons.

The importance of AVP neurons in circadian rhythms is now recognized (Mieda et al., 2015; Shan et al., 2020). AVP neuron-specific disruption of *Bmal1* using the Cre-lox system lengthened the period of circadian behavior and uncoupled activity onset and offset in constant darkness (DD), which was associated with lengthening of circadian rhythms in the dorsal area of the SCN (Mieda et al., 2015). At the cellular level, the circadian rhythms in AVP-*Bmal1* KO SCN neurons increased phase variance. These results suggested that the coupling of the E and M oscillators and/or the strength of circadian oscillation was attenuated by a loss of the AVP signaling. In this respect, AVP receptors, *V1a* and *V1b*, were suggested to have different roles in the coupling along the anteroposterior SCN (Bedont et al., 2018). Mice with disruption of *Bmal1* or overexpression of PER2 specifically in *Neuromedin S* (*Nms*) neurons exhibited deteriorated circadian behavioral rhythms under DD (Lee et al., 2015). Because NMS expression was restricted to the SCN, this approach provides an attractive tool for understanding the cell type-specific functions of SCN neurons.

Mice with *Bmal1* disruption specifically in *Avp* neurons (AVP-*Bmal1* KO) showed longer circadian behavioral rhythms and reduced *Avp* expression in the SCN (Mieda et al., 2015). This behavioral phenotype might be due to the reduction of neuropeptides in the SCN of AVP-*Bmal1* KO mice. Whereas, Smyllie et al (2016) demonstrated that deletion of *CK1εTau* alleles in *CK1εTau* mutant mice specifically in *dopamine 1a receptor* (*Drd1a*) expressing neurons (producing 24-h *Drd1a* cells and 20-h non-*Drd1a* cells) restored the circadian period in the majority of mice without attenuation of neuropeptide expression in the SCN. Because Drd1a expressing cells overlap with both AVP and VIP, *Avp* cells alongside non-*Vip Drd1a* cells and non-*Vip Nms* cells are critical for circadian pacemaking in the SCN (Smyllie et al., 2016). Such intersectional approaches have shown that ensemble period and rhythm stability are emergent properties of the SCN circuit, regulated by contributions from distinct cell groups. For example, the VIP-VIP-receptor cellular axis has been proposed as a pacemaking hub of the SCN circuit (Patton et al., 2020).

Although the importance of neuropeptidergic signaling in the SCN was identified, the regulation of transcription of these neuropeptides is also important. The transcription factor ZFHX3 regulates neuropeptidergic signaling by controlling the expression of both ligand- and receptor-encoding genes via circadian-regulated AT motifs located on their promoter regions (Parsons et al., 2015). Consequently, an induced *Zfhx3* missense mutation downregulated VIP and GRP expression in the SCN and shortened circadian period. Similarly, *Vax1* and *Six6* are transcription factors necessary for normal SCN development, neuropeptide expression in the SCN and normal circadian function (Clark et al., 2013; Pandolfi et al., 2020). These studies indicate that a transcriptional axis (AT motifs), itself under circadian control, nevertheless determined the robustness of SCN circadian rhythms and the output of the clock (AT-mediated transcription), thereby becoming an input to the clock and further stabilizing the system.

Immunohistochemistry has revealed that SCN cells express several other peptides or neurotransmitters such as calbindin, angiotensin II, and neurotensin, etc. (Abrahamson and Moore, 2001). Indeed, the complexity of neuropeptidergic signaling axes within the SCN has been revealed by single-cell RNA sequencing approaches (Morris et al., 2021; Wen et al., 2020). Furthermore, mass spectrometric method revealed that electrical stimulation of the retinohypothalamic tract induces release of several neuropeptides (Atkins et al., 2018) that hypothetically may function at the SCN neuronal circuit level.

In addition to these neuropeptides, it has long been known that almost all SCN neurons express the inhibitory neurotransmitter, gamma-aminobutyric acid (GABA) (Abrahamson and Moore, 2001; Card and Moore, 1984). Although several important papers related to GABA were reported in the past 25 years, the functional roles of GABA in the SCN are still debatable. It has been reported that GABA is a synchronizer of cellular circadian rhythms in the SCN. This was shown by measuring spontaneous firing rhythms in dispersed cell culture and applying GABA every 24 hours (Liu and Reppert, 2000) with the result that GABA entrained desynchronized SCN cells in culture to the same circadian phase. However, other studies suggest that GABA is a destabilizer or has no effect on circadian rhythms in the SCN (Aton et al., 2006; Freeman et al., 2013; Ono et al., 2019). Apparently, the role of GABA changes depending on the state of the SCN networks (Evans et al., 2013). When cellular circadian rhythms show wider phase distribution as seen in a long-day photoperiod, GABA works as a synchronizer, but when the SCN has a narrow phase distribution imposed by a short-day photoperiod, GABA works as a de-synchronizer. Thus, in a study of spontaneous firing rhythms after a 6-hours phase delay light schedule, the bimodal peak phases were observed in both dorsal and ventral SCN (Albus et al., 2005). Importantly, they gradually re-synchronized within 6 days. However, they showed unimodal patterns of firing rhythms with different peak phases between dorsal and ventral SCN with the application of the GABAA receptor antagonist, bicuculline, indicating that GABA is necessary for coupling between dorsal and ventral circadian rhythms in the SCN. Day-length modulates chloride homeostasis in the SCN by Cl^-^ transporters, KCC2 and NKCC1 (Myung et al., 2015; Rohr et al., 2019). The functions of GABA in the SCN therefore may depend on the state of the SCN network. In keeping with the notion that the state of the SCN changes over time, it has been reported that SCN neurons enter a state of depolarization block (Belle et al., 2009) though the generality of this phenomenon waits to be determined in the further studies.

#### IV D 3 Intracellular signaling

Timing information provided by extracellular signals modulates cellular functions via second messengers, such as cAMP or Ca^2+^. Intracellular Ca^2+^ showed clear circadian rhythms in individual SCN neurons (Ikeda et al., 2003a). Circadian rhythms of Ca^2+^ in the SCN continued under tetrodotoxin application. These results indicated that circadian Ca^2+^ rhythms were not regulated by the neuronal network, but instead depend on intracellular oscillatory mechanisms. Other groups have since demonstrated that circadian Ca^2+^ rhythms are regulated by both network and intracellular oscillatory mechanisms (Brancaccio et al., 2013; Enoki et al., 2012; Noguchi et al., 2017). Ca^2+^ has a variety of cellular functions, and Ca^2+^ flux is required for circadian rhythms of *Per1* expression in the SCN (Lundkvist et al., 2005). For example, application of voltage-gated Ca^2+^ channel antagonists into the culture medium reduces the amplitude of *Per1-luc* rhythms in the SCN. Intracellular Ca^2+^ would work for input as well as output from the transcription-translation feedback loop (TTFL) in the SCN. Intracellular cAMP is also crucial for circadian rhythms in the SCN. Pharmacological manipulation of intracellular cAMP modulates the amplitude, phase, and period of cellular circadian *Per1-luc* rhythms in the SCN (O’Neill et al., 2008) suggesting that the TTFL drives circadian rhythms of intracellular cAMP and its rhythms in turn regulate TTFL oscillation via cyclic AMP/ Ca^2+^ regulatory elements (CREs) in the *Per* genes (Travnickova-Bendova et al., 2002). Thus, circadian rhythms of cytosolic events reciprocally interact with the TTFL oscillation, output again becoming input to stabilize circadian rhythms in individual SCN cells.

#### IV E E and M oscillators: a tale of oscillator phase and location

In the classical two-oscillator model for nocturnal rodents of Pittendrigh and Daan (Pittendrigh and Daan, 1976) evening (E) and morning (M) oscillators are responsible for activity onset and offset, respectively. The E oscillator is synchronized to dusk, whereas the M oscillator is synchronized to dawn. Pittendrigh and Daan proposed a dual oscillator model to explain two mutually related phenomena associated with entrainment; holding stable phase-relations of the activity onset and end to light-dark (LD) cycles (ψLD), and adapting the behavioral rhythm to continuously changing photoperiod throughout the year. This dual oscillator model considers a key function of the circadian timing system: stable entrainment to the steadily changing photoperiods around the year in temperate zones. Equally important is the mystery of how a particular duration photoperiod (e.g. 14:10 vs 10:14) is encoded differentially in the spring and fall. The model assumes the evening (E) oscillator drives the onset of an activity band and the morning (M) oscillator which regulates the end of an activity in nocturnal animals. The concept is still useful and continues to draw attention, but its interpretation and meaning has shifted.

Several paradigms yield data that are consistent with the E and M concept, although taken together, they do not support the hypothesis that there is a specific location, across paradigms, for two stable distinct populations (Evans and Schwartz, 2023). There are populations of SCN neurons that oscillate stably in antiphase (Figure 2B). As noted above, antiphase oscillations of clock gene expression were observed in the left vs. right SCN, anterior vs. posterior, and core vs. shell of behaviorally split hamsters, suggesting again that in this protocol, these SCNs bear E and M oscillators respectively (de la Iglesia et al., 2000; Jagota et al., 2000; Ohta et al., 2005; Tavakoli-Nezhad and Schwartz, 2005; Yan et al., 2005; Zlomanczuk et al., 1991).

Photoperiod affects the pattern of rhythmic clock gene expression, providing insight into E and M oscillators (Johnston, 2005; Messager et al., 2000; Steinlechner et al., 2002). Inagaki et al. examined the circadian *Per1* expression rhythm in the coronal SCN slice of mice exposed to three different photoperiods and found a fixed phase-relation between the peaks of *Per1* rhythms and behavioral phase markers regardless of photoperiod (Inagaki et al., 2007). The circadian peak in the anterior SCN was locked on the activity onset and the peak in the posterior SCN was locked on activity termination. Cell level analyses revealed corresponding clusters of oscillating cells in the anterior and posterior SCN. These findings have been confirmed (Evans et al., 2011) and extended by using the SCN horizontal slice (Yoshikawa et al., 2017).

### IV F Oscillatory phenomena in clock gene deficient mouse SCN

Real-time recording of transcriptional oscillations in individual SCN cells has provided paradoxical results. Mice with disruption of one (*Bmal1*) or two closely related circadian clock genes (*Cry1/Cry2* or *Per1/Per2*) show arrhythmic behavior in DD (Bae et al., 2001; Bunger et al., 2000; van der Horst et al., 1999; Vitaterna et al., 1999; Zheng et al., 2001). However, transient circadian bioluminescence rhythms can be detected in SCN slices from these clock gene-deficient mice (Ko et al., 2010; Maywood et al., 2011; Maywood et al., 2013; Ono et al., 2013). The period of circadian rhythms of these slices is shorter compared with control mice. Interestingly, the circadian period of PER2::LUC rhythm in the neonatal SCN of the *Cry1/Cry2* KO mice was very short (ca. 16h) immediately after birth. The period was rapidly lengthened during the postnatal period to reach near 24 h by postnatal day 7. Since these rhythms were abolished with application of the sodium channel blocker TTX or an adenylyl cyclase inhibitor, neuronal networks in the SCN appear critical for the generation of these rhythms (Ono et al., 2013, 2016). On the other hand, some weak rhythms were also observed at the single cell level in dispersed SCN culture from *Cry1/Cry2* and *Bmal1* KO mice (Ko et al., 2010; Ono et al., 2013). Their period was widely distributed as compared with control mice and showed stochastic rhythms. It is still unclear how these rhythms are generated in single SCN cells. In mammals, redox oscillation with circadian period length have been observed in red blood cells which lack a nucleus (O’Neill and Reddy, 2011) and in the SCN (Edgar et al., 2012). This redox oscillation regulates neuronal excitability through a K^+^ channel in the SCN (Wang et al., 2012). Plausibly, TTFL-independent oscillatory mechanisms may exist in individual SCN cells from clock-deficient mice which allows the cells to express overt rhythms.

### IV G Functional roles of astrocytes in the SCN

The potential role of astrocytes in the SCN has been a subject of interest for some time (Serviere and Lavialle, 1996), with a focus on the modulation of synaptic signaling from the retinohypothalamic tract. More recent studies have been facilitated by two technical advances; the development of circadian reporters and the acquisition of genetic access to astrocytes. A brain-wide role for the astrocytic clock was indicated by the disruption of activity rhythms following global deletion of *Bmal1* from astrocytes (Barca-Mayo et al., 2017). More specifically to the SCN, the discovery that astrocytes from the cerebral cortex exhibit circadian rhythms of bioluminescent gene expression when held in dispersed culture (Marpegan et al., 2011; Prolo et al., 2005) was followed by the demonstration of both *Bmal1*- and *Cry1*-driven rhythms of bioluminescence by astrocytes in intact SCN slice cultures (Brancaccio et al., 2019; Tso et al., 2017). Intriguingly, SCN astrocytes also express pronounced rhythms of intracellular calcium ([Ca^2+^]_i_ but they peak in circadian night, in antiphase to neuronal [Ca^2+^]_i_ rhythms, which peak in circadian day when neurons are electrically and metabolically active (Brancaccio et al., 2017). This suggests that the clocks of SCN astrocytes and neurons operate in a mutually reinforcing circuit based on antagonistic interactions. Importantly, when the cell-autonomous circadian period of astrocytes is altered by intersectional genetic means in mice, the period of the behavioral activity rhythm changes accordingly. SCN astrocytes therefore act as circadian pacemakers to a functional SCN circuit. Furthermore, AAV-mediated astrocyte-specific expression of *Cry1* in *Cry*-null SCN can initiate circadian rhythms of gene expression and neuronal [Ca^2+^]_i_ in previously arrhythmic slices, and initiate circadian behavioral rhythms *in vivo* (Brancaccio et al., 2019). Thus, the cell-autonomous clock of SCN astrocytes is sufficient to direct circadian time-keeping in an otherwise clockless mouse.

Their reciprocally supportive interactions mean that circadian-competence in either astrocytes or neurons is sufficient to drive the SCN circuit. The question arises, therefore, as to how their contributions differ. Overall, neurons are more potent, insofar as they exert their actions on SCN period and the initiation of rhythms more rapidly and although astrocytes can slow SCN period to the same extent as can neurons, they are less able to accelerate it (Patton et al., 2022). Furthermore, whereas chemogenetic activation and inhibition of neurons can, respectively, delay and advance the phase of the SCN oscillation, similar manipulation of astrocytes is without effect, indicating that determination of SCN phase is reserved for SCN neurons, which receive the relevant cues via their innervation from the retina and midbrain. The principal role of astrocytes is to contribute to steady-state oscillation: its period and amplitude, but how might this be mediated? Astrocytes release a variety of gliotransmitters, including glutamate and ATP (Bazargani and Attwell, 2016) and cortical astrocytes release ATP in a circadian manner (Marpegan et al., 2011). In the SCN, extracellular levels of glutamate ([Glu-]e) oscillate with a peak in circadian night and with a waveform that maps on to the rhythm of astrocytic [Ca^2+^]_i_ suggesting that astrocytes are the source of this [Glu-]e in the GABAergic, i.e., non-glutamatergic, neuronal circuit (Brancaccio et al., 2017). Furthermore, pharmacological manipulation of [Glu-]e and glutamatergic signaling via ionotropic receptors containing the NR2C subunit compromise circadian time-keeping in the SCN slice. This supported a model whereby glutamate released by astrocytes in circadian night causes presynaptic depolarization of SCN neurons, leading to an increase in tonic GABA release and consequent suppression of neuronal activity. How SCN neurons in turn regulate the astrocytic clock is not known, although cortical astrocytes can be synchronized by co-culture with SCN slices (Prolo et al., 2005) and so paracrine neuropeptidergic cues such as VIP that sustain SCN oscillations (Maywood et al., 2011) may be involved (Marpegan et al., 2009) (Figure 2D).

### IV H Development of circadian systems in the SCN

#### IV H 1 Transcriptional specification of the SCN

The SCN undergoes neurogenesis, becomes an identifiable nucleus late in gestation, and continues to develop during the early postnatal period in nocturnal rodents (Altman and Bayer, 1978; VanDunk et al., 2011). Development proceeds in a region-specific manner, with the SCN core expressing VIP, GRP and calbindin (hamsters) 1-2 days earlier than the SCN shell expresses AVP (Antle et al., 2005; Carmona-Alcocer et al., 2020; Okamura et al., 1983). While the majority of the SCN neurogenesis is completed during the peri-natal period, some persists into adulthood (Mohr et al., 2017).

A number of transcription factors and signaling molecules play key roles in SCN development before and after neurogenesis and settling of the SCN, providing a genetic blueprint for the SCN. Among them, *Sonic hedgehog* (*shh*) is expressed before SCN neurogenesis and is critically involved in the formation of hypothalamic nuclei including the SCN via the secretion of the lipid-modified polypeptide morphogen (Alvarez-Bolado et al., 2012; Shimogori et al., 2010). Furthermore, a number of sequentially expressed transcription factors are involved in the neurogenesis and formation of the SCN. The expression of most of these early markers is transient, starting before the onset of SCN neurogenesis and ending before its completion (Bedont and Blackshaw, 2015; Shimogori et al., 2010), providing useful markers of SCN development. *Vax1* and *Rax* are transcription factors required for formation of the SCN (Pandolfi et al., 2020). *Sine oculis-related homeobox family transcription factor 3* and *6* (*Six3* and *Six6*) are expressed before SCN neurogenesis and a *LIM homeodomain transcription factor 1* (*Lhx1*) is expressed at the starting time of SCN neurogenesis. All of these transcription factors continue to be expressed in the SCN throughout the lifespan (Bedont and Blackshaw, 2015; Shimogori et al., 2010). Thus, the roles of these transcription factors are not limited to the formation of the SCN (VanDunk et al., 2011). Importantly, congenital deletion of *Six3* using *Nestin*-Cre results in a loss of SCN specification, and the absence of AVP, RORα and LHX1 in the suprachiasmatic region. The loss of AVP and RORα expression by deletion of *Six3* is SCN-specific, since these markers are expressed in other brain areas in *Six3*-deficient mice (VanDunk et al., 2011). Deletion of *Lhx1* in the developing SCN results in reduction of neuropeptides enriched in the SCN, such as AVP, VIP and GRP (Bedont et al., 2014; Hatori et al., 2014) and results in phenotypes similar to VIP knockouts, such as desynchrony of SCN cellular rhythms, reduced coupling among cellular oscillations and deterioration in circadian behavior rhythms (Aton et al., 2005; Harmar, 2003; Maywood et al., 2006). However, circadian rhythms are more strongly affected by deletion of *Lhx1* than by deletion of VIP, both *in vivo* (behavioral rhythms in DD) and *ex vivo* (neuronal activity in the SCN slice), suggesting *Lhx1*-specific and VIP-independent functions of *Lhx1* in the SCN. Indeed, *Lhx1* participates in regulation of sleep/wake rhythms and circadian resistance to fever (Bedont et al., 2017). Similarly, deletion of Zfhx3 prevents development of the SCN (Wilcox et al., 2021).

Astrogliogenesis in the SCN follows neurogenesis, as in other brain areas. Glial fibrillary acidic protein (GFAP), a marker protein of astrocytes, is detected at embryonic day (E) 20 (E20) in rat SCN. The expression increases at postnatal day (P) 3-4 (P3-P4) and again further at P20-P25 (Munekawa et al., 2000). The number of astrocytes increases in parallel with the extent of RHT innervation into the SCN in both hamsters and rats (Lavialle and Serviere, 1995; Munekawa et al., 2000). Rats undergoing bilateral eye enucleation immediately after birth do not show the dramatic increase in GFAP immunoreactivity at P20-25; their GFAP stays at low level even in adults (Munekawa et al., 2000). It is not the density of RHT terminals that matters for GFAP immunoreactivity, however, but their activity. GFAP immunoreactivity remains very low in rats kept in DD after birth, but the density of RHT terminals in the SCN does not differ from that in rats kept in LD. In contrast, the GFAP intensity increases when rats reared in DD are moved to LD lighting, and vice versa. Furthermore, *pituitary adenylate cyclase activating polypeptide* (PACAP), a neurotransmitter of RHT, but not glutamate, increases the length and number of astroglia in primary culture of hypothalamic astrocytes (Ikeda et al., 2003b). These findings suggest that postnatal development of astroglia in the SCN is reversible and dynamically regulated depending on the environmental light signals transmitted via the RHT.

#### IV H 2 An entrainable circadian clock is present in the developing SCN

Several approaches have been used to demonstrate the presence of a functional oscillator in the developing SCN. One method is to rear offspring in conditions where postnatal environmental influences are minimized (e.g., by rearing in constant darkness, or cross-fostering) and observe rhythms postnatally. This work shows that maternal entrainment starts prenatally and continues during the early postnatal period (Davis, 1997; Ohta et al., 2003; Reppert et al., 1984a; Reppert and Schwartz, 1986; Sasaki et al., 1984; Yamazaki et al., 2005). Multiple factors likely contribute to maternal entrainment of the developing offspring both pre- and postnatally, including rhythms in nutrients, hormones and temperature. Chemical signals that cross the placenta or physical signals such as activity/movements and body temperature are possible entraining time cues from mothers. Clock gene expression rhythms in the pups’ SCN revealed only a few cycles of exposure to periodic absence of the dam are enough to entrain pups’ rhythms when this manipulation occurs early in postnatal development (Yoshikawa et al., 2013).

A second method is to demonstrate prenatal entrainment is to directly detect rhythms in the developing SCN (Reppert and Schwartz, 1983). The earliest detection and start of oscillation during development have been reported by several labs (Ansari et al., 2009; Ohta et al., 2003; Shimomura et al., 2001; Sladek et al., 2004; VanDunk et al., 2011). and the results vary depending on the genes (*Per1, Per2, Cry1, Cry2, Bmal1, Cloc*k etc.), species (rats, mice or hamsters), detection techniques (mRNA or protein). It has also been reported that some of the genes related to neurodevelopment and cell-to-cell signaling showed circadian rhythms of transcription in the fetal SCN (E18-19), although clock genes did not (Greiner et al., 2022). This literature is covered in several recent reviews (Carmona-Alcocer et al., 2018; Sumova and Cecmanova, 2020).

Third, real-time imaging and reporter techniques enabled continuous monitoring of circadian rhythms from individual SCN cells as well as tissue explants. These techniques revealed the circadian oscillation of clock gene expression from the time of SCN neurogenesis (Carmona-Alcocer et al., 2018; Landgraf et al., 2015; Wreschnig et al., 2014). PER2::LUC was detected as early as E13.5, but explants at this age did not develop circadian rhythmicity *ex vivo*. A circadian PER2::LUC rhythm was detected in explants collected at E14.5 and afterwards (Carmona-Alcocer et al., 2018). Importantly, the study demonstrated that some extrinsic factor(s) starts the circadian rhythms at the very narrow time window during the development of fetal SCN, because the rhythm appearing at E14.5 was already synchronous at a time when the SCN expresses no known molecules for rhythm synchrony, such as VIP/AVP and their receptors. On the other hand, resetting of fetal SCN by the culturing process was reported by (Landgraf et al., 2015), even when peripheral tissues from the same animals were not reset by culturing.

#### IV H 3 Development of circuits within the SCN

*In vivo*, signals from the mother normally act on the entire SCN, and impinge on individual fetal SCN neurons to set their phase, generating a synchronized population. Several molecules are involved in synchronous rhythm expression in adult SCN (noted above), but fetal SCN can exhibit synchronous circadian rhythm much earlier than the expression of these potential synchronizing molecules (Carmona-Alcocer et al., 2018). How such a loose population of uncoupled oscillators can maintain coherent rhythmicity in the virtual absence of synapses and coupling mechanisms is a true mystery (Figure 2D).

The SCN exhibits robust and stable rhythms from the late gestational period throughout the life span, which however, does not necessarily mean that the circuit for the synchronous cellular rhythms stays the same thorough life. When the adult type SCN circuits fully develops is unknown. Synaptic as well as non-synaptic interactions function to synchronize cellular oscillations in the neonatal SCN. Diffusible factors from neonatal SCN synchronize cellular oscillation leading to coherent PER2::LUC rhythms in the SCN (Maywood et al., 2011). As in adults, tetrodotoxin desynchronizes the cellular rhythms of neonatal SCN (Ono et al., 2013; Yamaguchi et al., 2003). In adults, *Cryptochrome (Cry1)* and *Cry2* double-KO mice become behaviorally arrhythmic in DD (van der Horst et al., 1999; Vitaterna et al., 1999) and the SCN of CRY double-KO adult mice do not exhibit coherent circadian rhythms in clock gene expression (Albus et al., 2002; Kume et al., 1999; Okamura et al., 1999). Nevertheless, neonatal SCN of *Cry1/Cry2* double-KO mice can exhibit robust synchronized circadian rhythm in clock gene expression, spontaneous discharges, and intracellular Ca^2+^ through VIP signaling. This rhythmicity is gradually lost around the 3^rd^ postnatal week (Enoki et al., 2017; Honma, 2020; Ono et al., 2013). VIP is known to function as a synchronizer of cellular rhythms in the SCN (Maywood et al., 2011) and to form specific network in the SCN (Patton et al., 2020) for regulating behavior rhythms. This VIP function for rhythm synchronization is completed during development and once the VIP-dependent circuit is developed, loss of VIP neurons in adulthood does not disrupt behavior rhythms (Mazuski et al., 2020). During development, VIP gene expression is rhythmic, but the rhythmicity is masked by LD cycles in adulthood (Ban et al., 1997). Environmental light conditions are also an important factor for the circuit development.

## V Questions for the coming 25 years: the future of circadian time

### V A The cell-autonomous TTFL clock

Even with the conceptual breakthroughs of the past 25 years, which commonly resulted from technical developments, major questions about SCN structure and function remain. In considering cell-autonomous timekeeping, the TTFL model provides a powerful platform to understand “the clock” but we do not yet understand the balance between transcriptional and post-transcriptional regulation in generating rhythmic protein expression. We may speculate about serial delays adding up to a ∼24 hours process, but what are these delays, and are the mechanisms that introduce them qualitatively different from the more common cell biological processes, i.e., is there a particular circadian cell biology or are the component parts already known? Moreover, the linear concept of fixed temporal domains, each stage running sequentially into the next, may be misleading. Rather, any individual cellular process that contributes to time-keeping may occur at all phases of the cycle, and oscillation arises from subtle changes in the relative balance of these activities, each change tipping the likelihood of temporal progression. Such a clock mechanism therefore lacks internal boundaries, but, if that is so, what pushes it forwards and what stops it running backwards? In addition, the TTFL may not be the final word: the transient oscillations in BMAL1- and CRY-deficient SCN hint at additional mechanisms to define a ∼24 hours interval. This may be especially important in the developing SCN, when neuronal immaturity may push to the fore a deeper-seated, primitive timer, such as that active in anucleate red blood cells (Rey and Reddy, 2013) and sensitive to intercellular cues. Indeed, perhaps such a transiently resonant cytosolic system may continue to determine the rate and direction of the TTFL once the TTFL is established developmentally. It is more likely that in this extra-TTFL domain, rather than in the TTFL itself, that additional components of the molecular clockwork remain to be discovered.

Taking the TTFL at face value, many stylized diagrams across the literature illustrate protein blobs moving through cellular compartments alone and/ or in complex and associating with DNA. The experimental evidence in support of such models arises predominantly from observing the behavior of over-expressed proteins, the biochemical analysis of protein complexes released from the constraints of their cellular setting, recombinant versions *in vitro*, and the application of atypical (i.e., transformed and immortalized) cell lines. While useful, such approaches fail to address how the endogenous proteins interact in their native cellular setting and how their behavior is meshed into the electrical activity of SCN neurons. Overcoming these shortcomings will require the application of advanced intracellular imaging techniques based on super-resolution methods and quantitative analyses, informed by structural biology and allied with new techniques affording reversible control of the properties of clock proteins and their local cellular setting. The application of synthetic biology should have a major part to play in this. What are the respective roles of different PER and CRY proteins in negative feedback, and are they active simultaneously or at different stages of the cycle and/ or with different targets? Only by pulling on the cogs and levers of the SCN time-keeper and exercising quantitative, predictable control of its emergent properties (period, phase, amplitude etc.), both in a dish and *in vivo*, can we convince ourselves that we understand how it works. And we should avoid the pitfall of talking about the TTFL. Perhaps there is heterogeneity of circadian clock oscillations in the SCN: are there TTFL variants between cell populations or does a one-size TTFL fit all?

### V B The TTFL in an excitable cell

The next level of SCN organization pivots around the relationship between the TTFL and neuronal activity. The daytime peak of electrical firing is a fundamental property of the SCN across species with very different habits: the SCN encodes solar time, not behavior. There are now clear indications of how the TTFL directs circadian changes in the expression of ion channels, receptors and metabolic enzymes to sustain the peak and nadir. As the power of genetic and bioinformatic technology increases, then so will these causal pathways and regulatory genetic elements be mapped comprehensively across the genomes of SCN cells. In doing so, it will be interesting to see the higher-level mechanisms that temporally co-ordinate these changes, and this may inform more general understanding of electrical-metabolic coupling in neurons elsewhere. Conversely, it has become clear that electrical firing sustains the TTFL, in part by activity-dependent regulation of *Per* gene expression, and so an output of the clock, elevated firing rate, becomes an input to it. This re-entrance is likely mediated by cytosolic cues, notably [Ca ^2+^]_i_, driven by electrical activity, and perhaps intrinsic circadian oscillations in cytosolic pathways contribute to circadian stability and amplitude by fine-tuning the system. Importantly, such recurrent feedback does not sit well with the viewpoint of linear sequences, as exemplified by the input-clock-output model.

This general interplay between electrical firing and the TTFL is central to retinal entrainment of the SCN. When light is experienced during circadian night, glutamatergic (and perhaps neuropeptidergic) cues from retinal ganglion cells activate electrically quiescent SCN neurons in the core, especially VIP and GRP cells. This will increase the metabolic rate of those cells and induce *Per* expression, thereby shifting their TTFL. In parallel, it will stimulate second order, neuropeptidergic signals to non-retinorecipient SCN cells, increasing their firing and resetting the *Per* cycle. Overall, the entire circuit is phase-shifted, but we have only a qualitative perspective on the transduction pathways and molecular mechanisms of light-induced clock resetting. This is an area where new methods applicable *in vivo* – bioluminescent and fluorescent imaging of genetically encoded neuropeptide and neurochemical sensors alongside cell-type specific reporters of gene expression and cellular activity will reveal the complex spatio-temporal phenotype of SCN resetting. Tests of causal mechanisms within that phenotype, both cell-autonomous and at the network level, will then rely on loss- and gain-of-function genetic and pharmacological approaches. With quantitative specificity at the levels of genes, signals and behavior, we need to identify the routes and mechanisms whereby photic information is transduced through the SCN to re-direct circadian time. Related to this, we still do not understand the circuit-based plasticity whereby the SCN responds to the annual changes of photoperiod, and thereby determines the duration of the nocturnal secretion of melatonin, the “agent of darkness”.

A related consideration is the effect of arousal-mediated, non-photic resetting by neuropeptidergic, serotonergic and dopaminergic signaling (Grippo et al., 2017). The cellular actions of non-photic input signals to the SCN are poorly understood, and so their interactions with photic control are even less clear. Conceptually, they represent a further example of output of the clock (behavioral state) having the capacity for it to become clock input, again breaking the dogma of serial system components. This system also has translational relevance, insofar as shift work inevitably dissociates the cycles of rest/activity and light/ dark, causing circadian misalignment and poor health. Better understanding of the non-photic regulation of the SCN clock, again examined *in vivo*, will be important in seeking strategies to mitigate these deleterious effects.

### V C The steady-state SCN oscillatory circuit

Consideration of resetting leads to the larger question of how the SCN operates as a coherent time-keeping circuit under steady-state, free-running conditions. By exploiting intersectional genetics, it has been possible to show how the cell-intrinsic properties of particular cell groups in the SCN can influence, even dominate, behavioral phenotypes. What we lack is an integrative and comprehensive understanding of how the cell-types actually do operate together. How do they establish the emergent properties of the SCN, especially its accuracy and robustness, from less accurate and less robust cellular clocks? For example, do all cellular populations in the SCN contribute equally to the generation of network-level circadian time, or do distinct groups act as special pacemakers, and is that because of their particular properties and/ or their abundance? Although the locations of cell bodies have been well described, we know much less about their functional connectivity and whether communication depends on synaptic, paracrine or other modes of communication. Some evidence exists for a small-world network in the SCN, but the molecular identities of functional nodes, and of hubs with enriched connectivity, have yet to be determined. In addition, although the progressive wave of TTFL activity across the SCN has been evident since the first slice bioluminescence imaging studies, its neurochemical basis and the nature of the information it encodes are not known. We do know that neuropeptide signaling is important in the SCN, not least for synchronization of the circuit, but has that focus prevented us from thinking beyond the dichotomies of core and shell, VIP and AVP? The latest developments of single cell and spatial transcriptomics may allow us to develop a less biased view of SCN circuit structure by moving into new paradigms. A particularly intriguing possibility is that this may facilitate a better understanding of paracrine volume transmission in the SCN. To what extent do different ligand-expressing and receptor-expressing cell populations also express, respectively, the pre- and post-synaptic apparatus for loose, parasynaptic signaling as opposed to tight synaptic signaling? Whereas the latter may be ideal for rapid and acute information transfer, the former would be better suited to the slow and progressive signaling of circadian time across the SCN circuit. This raises the question of GABA, which has been proposed as a synchronizer or de-stabilizer or the circuit. Sustained activation of GABAergic synaptic tone is certainly inhibitory to the SCN, hyperpolarizing neurons and decreasing the amplitude of TTFL bioluminescence rhythms, but blockade of endogenous GABAergic signaling has minimal effect on the TTFL. It is therefore possible that ongoing GABAergic signaling is not really important for intra-SCN functions in the steady-state oscillation (but see below). Rather, its main role may be that of a synaptic signal to downstream targets as the critical relay for out-of-SCN function. If so, this raises very interesting questions about how an SCN neuron can operate as a volume signaler from its intra-SCN terminals but as a GABAergic synaptic signaler at its distal terminals. The same question could be applied to the neuropeptidergic axes of the SCN: to what degree are they intra- and extra-SCN in their roles and how do they achieve that?

Given that the SCN is an autonomous clock and everything required for time-keeping sits within the circuit, synaptic resolution from volumes of electron microscopic material could illustrate principles of how neural circuits fulfil their function. This is not possible with the analysis of similarly sized pieces of tissue from other brain regions, where function is not autonomous. It would also reveal the numbers, forms and locations of synaptic and extrasynaptic connectivity (e.g., gap junctions, volume transmission sites), and open the way to plot their ontogeny. How does the assembly of the SCN at a synaptic level lead to its autonomy of function as a timer, and then how do afferents from retina, brain stem etc. wire into that circuit? A further advantage of such reconstructions is that all cell types are included and so the relationships between SCN neurons and glia, local vascularization and ependymal lining can be viewed in the whole. The recent discovery of a portal system linking the SCN and organum vasculosum (Yao et al., 2021) has pointed to an entirely new route and targets for secreted SCN signals, and potentially restructures our understanding of brain communication pathways. The neural efferents may not be the sole means of signaling SCN time: what is the relationship between the SCN connectome and angiome, and what is the relationship between the SCN and the CSF? Are there additional pathways for the transmission of SCN circadian time that await discovery? Beyond the biology, the circadian properties of the SCN, with its output being akin to a sine wave, have attracted considerable attention from modelers, both for cell-autonomous and circuit-level organization (Pauls et al., 2016). Indeed, there is considerable work on intra-SCN networks involving back-and forth between biology and modeling work leaving a major question on how to test models against each other. Such opportunities arise, for example, when biological results are simulated in models using Kuramoto oscillators where amplitude, phase, period and connectivity are among the parameters manipulated. This is most relevant in describing and analyzing the emergent properties of the SCN: ensemble phase and period, rhythm amplitude and robustness, and the stable stereotypical phase dispersion across SCN sub-populations. The stereotypical wave of peak TTFL activity that flows across the SCN encodes information. Some links between ligand-and receptor-expressing cells (e.g., VIP and Prokineticin 2 and Prokineticin receptor 2) may constitute segments of this re-entrant spatio-temporal axis. Its overall topology and function, however, await investigation and model-based insights may be invaluable in such a task, although a challenge remains on how to test models against each other.

### V D SCN neurons and astrocytes as pacemakers: a cellular pas-de-deux?

The demonstration that SCN time-keeping persists when astrocytes are the only cells with a functional TTFL raises a series of questions, not least what TTFL-dependent signals emanating from the astrocytes convey circadian cues to SCN neurons, which are the ultimate arbiters of circadian behavior. Astrocytic control of extracellular levels of glutamate and GABA, allowing daytime neuronal activity is one proposed mechanism but their molecular and cellular underpinnings and how they relate to the nocturnal elevation of [Ca ^2+^]_i_ in astrocytes are not known. Nevertheless, this role for extra-synaptic signals echoes the importance of paracrine information transfer across the SCN. Even less is understood of how SCN neurons, reciprocally, signal circadian time to astrocytes. This may be neuropeptidergic, although the antiphasic rhythms of [Ca ^2+^]_i_ of neurons and astrocytes suggests the effect is inhibitory, in contrast to the stimulatory role of neuropeptides on SCN neuronal function. The development of methods to image and manipulate cellular functions and the extracellular milieu of the SCN will be necessary to provide answers. A broader question is whether the communication of time between neurons and astrocytes, seen in the SCN, is a more general model, applicable to other brain regions. Conventionally, the tri-partite synapse of pre- and post-synaptic neurons and enclosing astrocyte has been considered in the context of rapid (millisecond) synaptic signaling. But the SCN also operates on a different, far longer time-base with activities changing over hours. Nevertheless, it may well be the case that similarly slow, progressive state changes, for example transitions of sleep stage, may also be directed by astrocytic cues. The presence of circadian TTFL function in cortical astrocytes and its response to neuropeptidergic signals (Prolo et al., 2005) suggests that the system uncovered in the SCN may be more generally applicable. Another twenty-five year should be long enough to answer that.

### V E Timing the organism: circadian alignment and misalignment

Our knowledge of how the SCN co-ordinates the intrinsically generated circadian rhythms in other brain structures is largely limited to understanding the anatomy and neurochemistry of its efferent neural pathways, which remains incomplete. Neuropeptidergic and/ or GABAergic control of immediate neural targets may be readily explored, but multi-synaptic linkages introduce greater complexity. The implementation of new-generation trans-synaptic viral vectors, such as self-inactivating rabies variants (Ciabatti et al., 2020) offers great promise not only to map this complexity, but also to deliver genetically encoded reporters and effectors to monitor and manipulate the circuit nodes with spatial and temporal precision. These will be combined with sophisticated, remote monitoring of behavior and physiology because the ultimate test of our knowledge will be the successful linking of SCN outflow to outcomes *in vivo*: can we predict, with quantitative precision, how the animal will respond to particular manipulations of the SCN and its downstream circuits? What processes are necessary for circadian alignment and what are sufficient? We shall then be able to answer question such as “how does the SCN control the phase of rhythms in peripheral tissues”, and “how/ why does the phase of gene expression rhythms in peripheral oscillators and oscillators in brain areas outside the SCN lag 4 to 6 hours behind the SCN?”

Inevitably, initial progress will be made in the genetically tractable mouse, because manipulation or recording of some specific cells can identify the functional roles of these cells. However, broadening to others species with different temporal habits will provide a comprehensive view of conserved and locally adapted sub-systems, such as photoperiodism. This will inform the translation to the clinic of discoveries in circadian neurobiology. We need to know more about such mechanisms and the pathophysiology of circadian rhythm mis-alignment if the mechanistic advances are to bear fruit. In this regard, the multi-faceted morbidities associated with rotational shift-work can be viewed as the principal area of public health where circadian knowledge will have direct application. This is not, necessarily, to argue for a ban on such practices because round-the-clock operations are necessary for our emergency services and numerous industrial processes. Moreover, they can bring social and economic benefit to workers. Rather, circadian knowledge will inform how to mitigate the associated risks by establishing effective, targeted monitoring of health-status, the design of circadian-informed working patterns and the avoidance of poor eating habits and unhelpful self-prescribed medications. Circadian mis-alignment is a stress and should be managed as any other of the inevitable stresses of a life lived. The incorporation of circadian and sleep-related information alongside genomic information in major health data-bases will facilitate such evidence-based approaches. Such new technologies, applied at scale, will be the way to address such population-based circadian disease and deliver on the discoveries of the past 50 years.

## Figures and Tables

**Figure 3:**
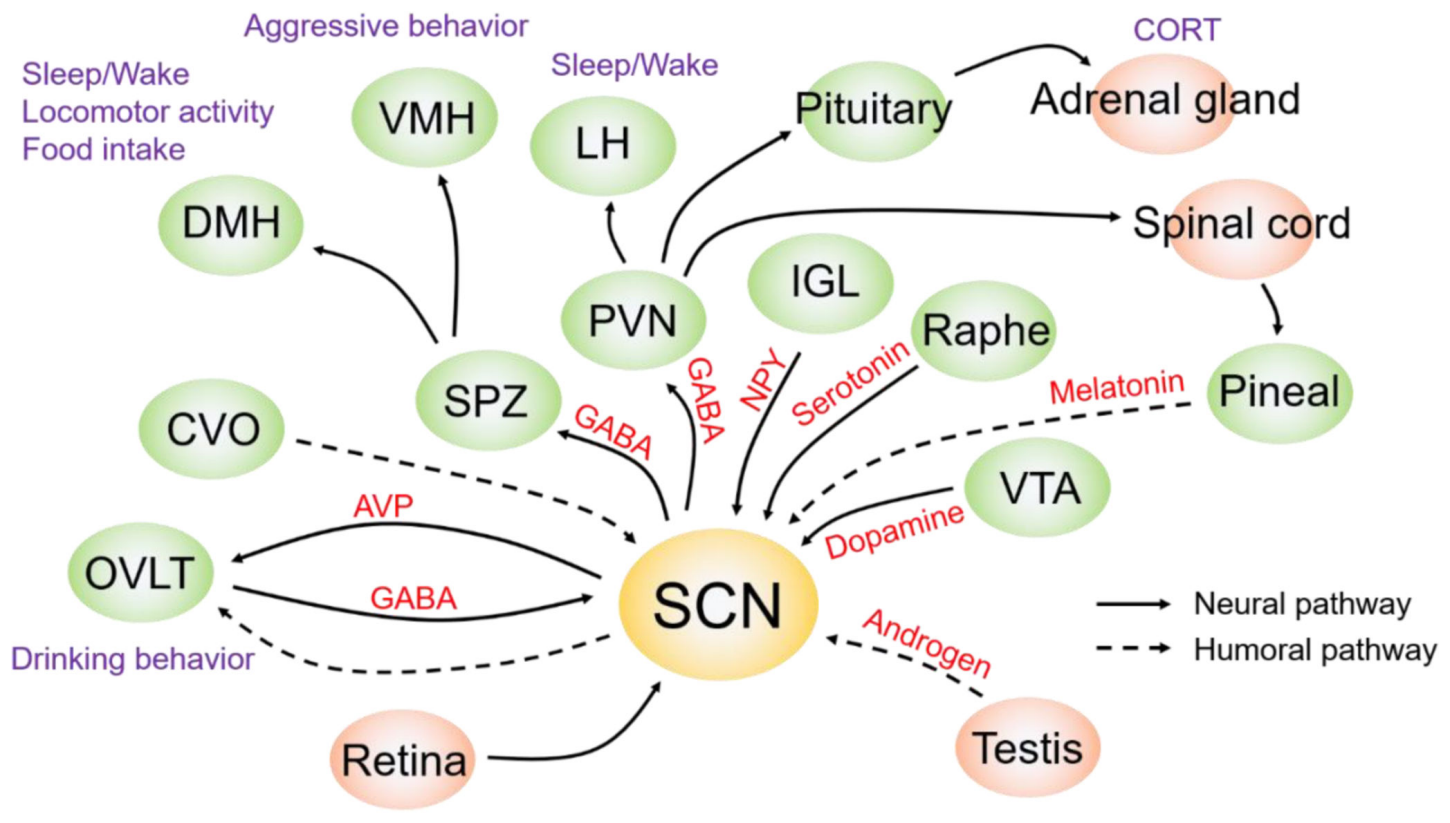
Circadian input and output pathways in the SCN Anatomical input and output pathways were reported in the first 25 years. During the second 25 years, functional input and output pathways were identified. Solid and broken arrows are neuronal and humoral pathways, respectively. Purple and red letters indicate output phenotypes and related molecules, respectively. Green and orange circles indicate input/output brain areas and non-brain areas, respectively. SCN: suprachiasmatic nucleus, OVLT: organum vasculosum laminae terminalis, SPZ: subparaventricular zone, PVN: paraventricular hypothalamic nucleus, CVO: circumventricular organs, DMH: dorsomedial hypothalamus, VMH: ventromedial hypothalamus, IGL: intergeniculate leaflet, VTA: ventral tegmental area, LH: lateral hypothalamus, CORT: corticosterone.
